# Supplementary material for: Neuraminidase B controls neuraminidase A-dependent mucus production and evasion
Source: PLoS Pathog. 2021 Apr 5;17(4):e1009158. doi: 10.1371/journal.ppat.1009158 (PMC8049478; doi:10.1371/journal.ppat.1009158)
Supplement: S1 Fig — To quantify sialic acid and mucus containing secretions in the URT of mice, retro-tracheal lavages were obtained from infant mice at day 5 p.i.. After collection, using a slot-blot manifold apparatus an immunoblot was performed to determine the amount of α-2,3 linked sialic acid present in different infections. There are 3 infection types, mock-infected, Type 23F::pilus-1 and ΔnanA,nanB::janus. Each infection type is shown for two animals and each shown in triplicate with individual animals denoted by the type of infection and replicate. Immunoblots were used for quantification by densitometric analysis. (DOCX) [file ppat.1009158.s001.docx]

**S1 Fig: Representative Immunoblot for detection of α2,3 linked sialic acid**


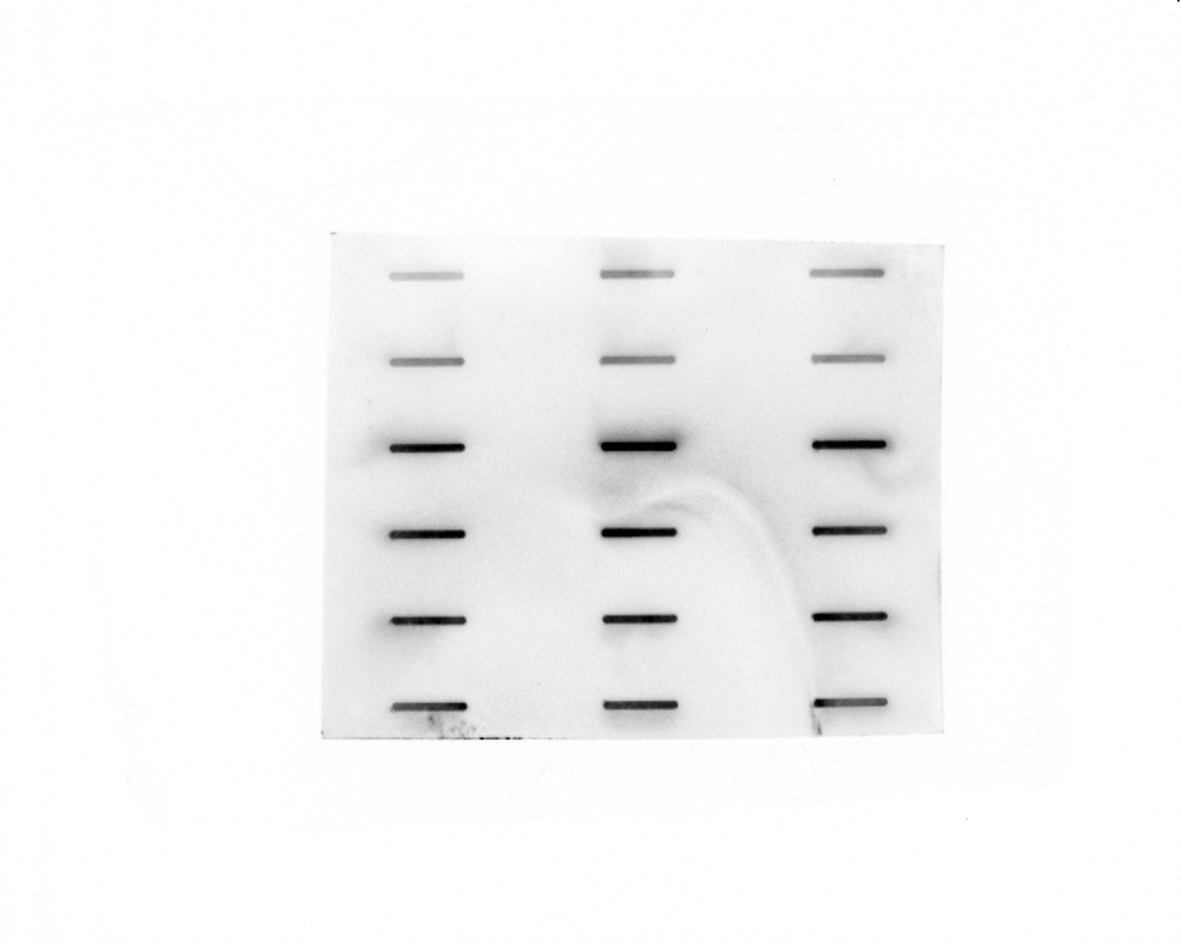


**Mock (1)**

**Type 23F::pilus-1 (1)**

**Type 23F::pilus-1 (2)**

**Δ*nanA*,*nanB*:*:*janus (1)**

**Δ*nanA*,*nanB*:*:*janus (2)**

**Replicate**

**(1)**

**Replicate**

**(2)**

**Replicate**

**(3)**

**Mock (2)**
