# Supplementary material for: Neuraminidase B controls neuraminidase A-dependent mucus production and evasion
Source: PLoS Pathog. 2021 Apr 5;17(4):e1009158. doi: 10.1371/journal.ppat.1009158 (PMC8049478; doi:10.1371/journal.ppat.1009158)
Supplement: S1 Table — Bacterial strains used in this study are outlined in S1 Table. Information includes the serotype, genotype, antibiotic resistance and the source or reference for the strain. (DOCX) [file ppat.1009158.s002.docx]

**S1 Table. Bacterial Strains used for the study.**

| **Strain**  ***Streptococcus pneumoniae*** | **Sero- and genotype** | **Antibiotic resistance** | **Source or reference** |
| --- | --- | --- | --- |
| P2406 | 4 (TIGR4), clinical isolate | Strep^r^ | 17 |
| P2408 | 23F, *ply::*janus | Kan^r^ | 27 |
| P2422 | TIGR4Δ*cps* | Kan^r^ | 17 |
| P2583 | TIGR4ΔpspC(cbpA) | Strep^r^ | 5 |
| P2499 | 23F, clinical isolate | Strep^r^ | 18 |
| P2588 | 23F::*pilus-1* (pilus-1 insertion) | Strep^r^ | 5 |
| P2613 | TIGR4 *nanB::*janus | Kan^R^ | This study |
| P2619 | TIGR4Δ*nanB* | Strep^R^ | This study |
| P2622 | 23FΔ*nanB* | Strep^R^ | This study |
| P2438 | TIGR4Δ*cps::cps* | Strep^R^ | 16 |
| P2623 | TIGR4Δ*nanB::nanB* | Strep^R^ | This study |
| P2613 | 23FΔ*nanB::nanB* | Strep^R^ | This study |
| P2605 | TIGR4Δ*nanA* | Strep^R^ | This study |
| P2634 | P2588*,* Δ*nanA* | Strep^R^ | This study |
| P2635 | P2588*,* Δ*nanA::nanA* | Strep^R^ | This study |
| P2636 | P2588*,* Δ*nanA, nanB::j*anus | Kan^R^ | This study |
| P2637 | P2588*,* *nanB::*janus | Kan^R^ | This study |
| P2641 | P2588*,* *nanB::nanB* | Strep^R^ | This study |
| P2539 | TIGR4Δ*mucBP* | Strep^R^ | This study |
| P2547 | TIGR4Δ*estA* | Strep^R^ | This study |
| P2503 | TIGR4Δ*bgaA* | Erm^r^ | 15 |
| P2460 | 23FΔ*bgaA* | Erm^r^ | 6 |
| P1519 | 23FΔ*strH* | Spec^R^ | 6 |
| P2642 | P2588*,* NanB*_D270A_* | Strep^R^ | This study |
