# Supplementary material for: Neuraminidase B controls neuraminidase A-dependent mucus production and evasion
Source: PLoS Pathog. 2021 Apr 5;17(4):e1009158. doi: 10.1371/journal.ppat.1009158 (PMC8049478; doi:10.1371/journal.ppat.1009158)
Supplement: S2 Table — Bacterial primers used in this study for strain construction are outlined in S2 Table. Information includes the gene target, primer name and Sequence (5’→3’) for the primer. (DOCX) [file ppat.1009158.s003.docx]

**S2 Table: Primers used for bacterial strain construction.**

| **Gene Target** | **Primer Name** | **Sequence (5’→3’)** |
| --- | --- | --- |
| P2619  Clean deletion *nanB* in Type 4 | 1. *nanB* upstream forward  2. *nanB* upstream reverse  3. *nanB* downstream forward  4. *nanB* downstream reverse | cctgggctggggaactaaattgttgg  ctcctttattttgttaaatcacctcttttattcatttttttcc  ggaaaaaaatgaataaaagaggtgatttaacaaaataaaggag  gctccttccataatctcatgcagatagcgc |
| P2621  *nanB::Janus-cassette* in Type 23F | 5. *nanB* upstream forward  6. *nanB* upstream reverse  7. *nanB* janus forward  8. *nanB* janus reverse  9. *nanB* downstream forward  10. *nanB* downstream reverse | gctatcatcagtaagtgggaaaaggagg  cacattatccattaaaaatcaaacggacctcttttattcatttttttccttctttcg  cgaaagaaggaaaaaaatgaataaaagaggtccgtttgatttttaatggataatgtg  ctcctttatttttatgcttttggacgtttagtacc  ggtactaaacgtccaaaagcataaaaataaaggag  ccttggattaagacataatgttcaccccaacg |
| P2622  Clean deletion *nanB* in 23F | 11. *nanB* upstream forward  12. *nanB* upstream reverse  13. *nanB* downstream forward  14. *nanB* downstream reverse | gctatcatcagtaagtgggaaaaggagg  tcctttattttgttaaatcacctcttttattcatttttttcc  ggaaaaaaatgaataaaagaggtgatttaacaaaataaaggag  ccttggattaagacataatgttcaccccaacg |
| P2623  Corrected mutant *nanB* in Type 4 | 15. *nanB* upstream forward  16. *nanB* downstream reverse | cctgggctggggaactaaattgttgg  gctccttccataatctcatgcagatagcgc |
| P2613  Corrected mutant *nanB* in 23F | 17. *nanB* upstream forward  18. *nanB* downstream reverse | gctatcatcagtaagtgggaaaaggagg  ccttggattaagacataatgttcaccccaacg |
| P2604  *nanA::Janus-cassette* in Type 4 | 19. *nanA* upstream forward  20. *nanA* upstream reverse  21. *nanA* janus forward  22. *nanA* janus reverse  23. *nanA* downstream forward  24. *nanA* downstream reverse | ggttactgacttcgtcagttctatccacaaccc  cacattatccattaaaaatcaaacggaattcatactgatcctc  gaggatcagtatgaattccgtttgatttttaatggataatgtg  cttctcttattgttctcttatgcttttggacgtttagtacc  ggtactaaacgtccaaaagcataagagaacaataagagaag  tcaacttcaactgttattcgtaagctcaatgac |
| P2605  Clean deletion of *nanA* in Type 4 | 25. *nanA* upstream forward  26. *nanA* upstream reverse  27. *nanA* downstream forward  28. *nanA* downstream reverse | ggttactgacttcgtcagttctatccacaaccc  cttctcttattgttctctcttaacactccgattcatactgat  atcagtatgaatcggagtgtt aagagagaacaataagagaag  tcaacttcaactgttattcgtaagctcaatgac |
| P2632  *nanA::Janus-cassette* in P2588 | 29. *nanA* upstream forward  30. *nanA* upstream reverse  31. *nanA* janus forward  32. *nanA* janus reverse  33. *nanA* downstream forward  34. *nanA* downstream reverse | cttcgccttgccgtaggtat  cacattatccattaaaaatcaaacggagagtttcatttgcc  ggcaaatgaaactctccgtttgatttttaatggataatgtg  gaattcttctcttattgttatgcttttggacgtttagtacc  ggtactaaacgtccaaaagcataacaataagagaagaattc  ccccattcttctgtctagcattttctac |
| P2634 Clean deletion of *nanA* in P2588 | 35. *nanA* upstream forward  36. *nanA* upstream reverse  37. *nanA* downstream forward  38. *nanA* downstream reverse | cttcgccttgccgtaggtat  cttctcttattgttctctcttaacactccgattcatactg  cagtatgaatcggagtgttaagagagaacaataagagaag  ccccattcttctgtctagcattttctac |
| P2635 Corrected mutant of *nanA* in P2388 | 39. *nanA* upstream forward  40. *nanA* downstream reverse | cttcgccttgccgtaggtat  ccccattcttctgtctagcattttctac |
| P2637  *nanB::janus* in P2588 | 41. *nanB* upstream forward  42. *nanB* upstream reverse  43. *nanB* janus forward  44. *nanB* janus reverse  45. *nanB* downstream forward  46. *nanB* downstream reverse | gctatcatcagtaagtgggaaaaggagg  cacattatccattaaaaatcaaacggacctcttttattcatttttttccttctttcg  cgaaagaaggaaaaaaatgaataaaagaggtccgtttgatttttaatggataatgtg  ctcctttatttttatgcttttggacgtttagtacc  ggtactaaacgtccaaaagcataaaaataaaggag  ccttggattaagacataatgttcaccccaacg |
| P2641 Corrected mutant of *nanB* in P2588 | 47. *nanB* upstream forward  48. *nanB* downstream reverse | gctatcatcagtaagtgggaaaaggagg  ccttggattaagacataatgttcaccccaacg |
| P2636 *nanB::janus* in P2634 | 49. *nanB* upstream forward  50. *nanB* upstream reverse  51. *nanB* janus forward  52. *nanB* janus reverse  53*. nanB* downstream forward  54. *nanB* downstream reverse | gctatcatcagtaagtgggaaaaggagg  cacattatccattaaaaatcaaacggacctcttttattcatttttttccttctttcg  cgaaagaaggaaaaaaatgaataaaagaggtccgtttgatttttaatggataatgtg  ctcctttatttttatgcttttggacgtttagtacc  ggtactaaacgtccaaaagcataaaaataaaggag  ccttggattaagacataatgttcaccccaacg |
| P2526  *mucBP::Janus-cassette* in Type 4 | 59. *mucBP* upstream forward  60. *mucBP* upstream reverse  61. *mucBP* janus forward  62. *mucBP* janus reverse  63. *mucBP* downstream forward  64. *mucBP* downstream reverse | ggttgggctagatctggttctgaagagttc  cattatccattaaaaatcaaacggataatgatacgcgttatagtcttcgt  acgaagactataacgcgtatcattatccgtttgatttttaatggataatg  ctaaaatctattatttttcatcttcttatgcttttggacgtttagtacc  ggtactaaacgtccaaaagcataagaagatgaaaaataatagattttag  gcctttatgctcaccaagaactgatttgggaagg |
| P2539  Clean deletion *mucBP* in Type 4 | 65. *mucBP* upstream forward  66. *mucBP* upstream reverse  67. *mucBP* downstream forward  68. *mucBP* downstream reverse | ggttgggctagatctggttctgaagagttc  gattctaaaatctattatttttcatcttctgttttgggaaccactgtttcctc  gttgaggaaacagtggttcccaaaacagaagatgaaaaataatagattttag  gcctttatgctcaccaagaactgatttgggaagg |
| P2537  *estA::Janus-cassette* in Type 4 | 69. *estA* upstream forward  70. *estA* upstream reverse  71. *estA* janus forward  72. *estA* janus reverse  73. *estA* downstream forward  74. *estA* downstream reverse | gacaggtcgtatgggtgagc  cacattatccattaaaaatcaaacggatgccatgattttctccttc  gaaggagaaaatcatggcatccgtttgatttttaatggataatgtg  ccccctatgctgaagttcaaactaagtccagagacctgggcccctttccttatgc  gcataaggaaaggggcccaggtctctggacttagtttgaacttcagcataggggg  agccctttgtttcgtgctgt |
| P2547  Clean deletion *estA* in Type 4 | 75. *estA* upstream forward  76. *estA* upstream reverse  77*. estA* downstream forward  78. *estA* downstream reverse | gacaggtcgtatgggtgagc  gctgaagttcaaactaagtcagtctctctttcatcactgccatgattttctcc  ggagaaaatcatggcagtgatgaaagagagactgacttagtttgaacttcagc  agccctttgtttcgtgctgt |
| P2642 Δ*nanB_D240A_* in P2637 | 79. *nanB* upstream forward  80. *nanB* forward with mutation  81. *nanB* reverse with mutation  82. *nanB* downstream reverse | gctatcatcagtaagtgggaaaaggagg  cgttatggtgggactcatgcctctaaaagtaagattaatattgcc  ggcaatattaatcttacttttagaggcatgagtcccaccataacg  ccttggattaagacataatgttcaccccaacg |
| P2613 *nanB::Janus-cassette* in Type 4 | 83. *nanB* upstream forward  84. *nanB* upstream reverse  85. *nanB* janus forward  86. *nanB* janus reverse  87. *nanB* downstream forward  88. *nanB* downstream reverse | cctgggctggggaactaaattgttgg  cacattatccattaaaaatcaaacggaattcatttttttcc  ggaaaaaaatgaattccgtttgatttttaatggataatgtg  ctcctttatttttatgcttttggacgtttagtacc  ggtactaaacgtccaaaagcataaaaataaaggag  gctccttccataatctcatgcagatagcgc |
