# Supplementary material for: Neuraminidase B controls neuraminidase A-dependent mucus production and evasion
Source: PLoS Pathog. 2021 Apr 5;17(4):e1009158. doi: 10.1371/journal.ppat.1009158 (PMC8049478; doi:10.1371/journal.ppat.1009158)
Supplement: S3 Table — Bacterial primers used in the study for quantitative RT-PCR are outlined in S3 Table. Information includes the gene target, primer name and Sequence (5’→3’) for the primer. (DOCX) [file ppat.1009158.s004.docx]

**S3 Table. Primers used for quantitative RT-PCR in this study.**

| **Gene Target** | **primer name** | **sequence (5’→3’)** |
| --- | --- | --- |
| *gapdh* | *gapdh* forward  *gapdh* reverse | ggtcgtcttgctttccgtcgtatc  gctttcatagctgcgttcacttcatc |
| *16S* | *16s* forward  *16s* reverse | acccgaagtcggtgaggta  ccaaatcatctatcccacctt |
| *nanA* | *nana* forward  *nana* reverse | cggtaggagcggtttctatg  gctaaaacaggagacgttccaa |
| *nanB* | *nanb* forward  *nanb* reverse | ggcaacactaggagctgttaatc  tcaatacttccttttgcgaggt |
| *nanR* | *nanr* forward  *nanr* reverse | agacttgattgacgaaatccaac  gatacgctcagcatcttcgat |
