# Supplementary material for: Neuraminidase B controls neuraminidase A-dependent mucus production and evasion
Source: PLoS Pathog. 2021 Apr 5;17(4):e1009158. doi: 10.1371/journal.ppat.1009158 (PMC8049478; doi:10.1371/journal.ppat.1009158)
Supplement: Attachment — Submitted filename: Plos Pathogens Response to Reviewers_JW_2_13_21.docx [file ppat.1009158.s005.docx]

To PLoS Pathogens Editors:

We thank the three Reviewers for their carefully attention to our manuscript and their thoughtful suggestions. Their comments were addressed in the attached revised version and point-by-point responses to their comments below (in red). We added new data in Figures IE and G, 6A and B and a new supplemental figure (S1).

The reviewers were generally very positive towards the manuscript. However meaningful concerns regarding the methodology used in mucus collection and the integrity of the mucus scaffold during experimentation require attention. It is important that the mucus model be validated prior to resubmission. Additionally, better explanation of the statistical tests chosen and clarity in the legends is requested.


Reviewer's Responses to Questions

**Part I - Summary**
Please use this section to discuss strengths/weaknesses of study, novelty/significance, general execution and scholarship.

Reviewer #1: This is an interesting paper that explores a relevant topic: how bacteria evades mucociliary clearance as a defense mechanism. The experiments conducted and discussed are relevant and significant. Overall, the paper is strong, with one major weakness in the characterization of the mucus samples, as outlined below.

See comments below

Reviewer #2: Comments for the authors of the PLoS Pathogens manuscript number PPATHOGENS-D-20-02578:
The authors of the PLoS Pathogens manuscript “Neuraminidase B controls neuraminidase A-dependent mucus production and evasion”, presents some very interesting findings related to host:pathogen interactions with Streptococcus pneumoniae. Specifically, this group has been using their recently-developed solid-phase adherence assay to evaluate Spn adherence as they study bacterial interactions with the host in the presence of mucus. This manuscript shows that the neuraminidase genes NanA and NanB both contribute to mucus evasion through removal of terminal sialic acids and that, interestingly, NanB controls NanA expression. This process works through NanB releasing sialic acid residues that then activate NanA activity. Ultimately, NanA performs the bulk of the sialic acid modifications, while NanB is required to get the process started. The group did an excellent job of identifying the specific contributions of NanA and NanB to this process, including demonstrating that this was not controlled by NanR. This well-designed, well-controlled study presents some interesting results that are described with appreciation for how these findings advance the field. There was only one minor typographical error that needs to be corrected, and that is listed below.

See comments below

Reviewer #3: This is an interesting and generally well written manuscript that addresses the long-standing question of what the biological function of NanB might be, given that all previously known contributions of pneumococcal neuraminidase are attributed to NanA. The experiments and data are for the main part logical and support the conclusions of the Authors. These are complex models and so it is not surprising that there is a lot of variability in some of the data. However, a range of statistical tests are applied, and the logic is not explained. This leaves one wondering if there is a reason or if this was just to achieve statistical significance. There are other relatively minor concerns regarding the clarity of legends and strain names, the interpretation of data and the conclusions drawn. A more in-depth discussion of what might occur in nanB negative isolates (especially those that also lack nanC) would be interesting. Those strains still appear to maintain the rest of the nanB operon – making this Reviewer wonder if they scavenge 2,7-anhydroneuraminic acid released by other bacteria.

See comments below

**Part II – Major Issues: Key Experiments Required for Acceptance**

 Reviewer #1: 1. Mucin methods are very non-specific. The nasal washes, while appropriate to test the question, as Spn colonized the nasal cavity, will capture many things in the mucus besides mucin. The washes are not well described. What is the concentration of the mucus in the washes? How many cells are captured in the wash? Are they removed?

We purposefully took a broad-view of the interactions of Spn with upper respiratory tract (URT) material, as our prior work (Binsker et al, *J Clinical Invest* 2020) established an important role for non-mucin components in Spn interactions with mucus. Therefore, we avoided the use of purified components, such as mucins, and instead used native, untreated human nasal fluid (hNF) and murine URT lavages (mNL) to delineate host-pathogen interactions. Our strategy and approach are laid out in the first paragraph of the discussion (lines 397-410).

mNLs obtained using PBS were pooled and then stored without further treatment at -20°C. hNF samples were too viscous to apply consistently and were sonicated at low power for one second (also see comment below about sonication) prior to pooling and storage at -20°C.

To control for sample to sample variation, we quantified total protein concentration and then adjusted samples to an equal protein concentration (10ug/100ul) with PBS for our assays using hNF. Additional detail about protein concentrations and equilibration were added to the methods in lines 635.

Using light microscopy, no intact cellular material was seen in either the mNLs or hNF as demonstrated by Fig. 1E, G, respectively.

2. Sonication will disrupt mucin polymers, causing the sample to not be native as characterized in the manuscript. Disruption of polymers will change the availability of the side chains, including the sialyated groups, for the Spn to act on enzymatically. Validation data demonstrating that sonication is not influencing the results is necessary.

The murine nasal lavages (mNLs) were not sonicated for our adherence and other assays. In Fig. 1A, we demonstrate that prolonged sonication does decrease Spn adherence to immobilized mNLs. For human nasal lavages, it was necessary to sonicate our samples for one second under low power (amplitude 8), because these samples were highly viscous and could not be aliquoted evenly. Figure 1A demonstrates that one second of sonication under these conditions has no significantly impact on bacterial adherence.

*3. In Figure 1E, there is no way to identify the faint blue dots as mucus particles. The panel needs a scale bar is needed for this panel. IHC for Muc5ac, as done in the later figures, is necessary here.*

As suggested, we added scale bars (10um) and performed additional microscopy (IHC) to show that Spn is interacting with Muc5ac-containing material found within human nasal fluid (added to Fig. 1, panel G). Alcian-blue, which stained globules bound by bacteria, is a well-recognized stain for mucopolysaccharides.

*Reviewer #2: None required.*

*Reviewer #3: None*

***Part III – Minor Issues: Editorial and Data Presentation Modifications*** *Please use this section for editorial suggestions as well as relatively minor modifications of existing data that would enhance clarity.*

*Reviewer #1: 1. Table 1 is confusing, especially the second part with all the foot notes. Why were some mutations only tested in one strain? Some of the data suggests that the strain is important. This might be better represented as a bar graph.*

We screened candidate genes in the Type 4, TIGR4 isolate (except for *strH* as we were unable to construct the mutant in strain TIGR4). If we observed an effect of the mutation in strain TIGR4, we then moved forward with that gene and tested/confirmed its effect in an unrelated isolate (Type 23F background). We added the additional information into the manuscript (line 163) to help explain the table and our screening strategy.

*2. In line 159, do you intend to refer to Figure 2C?*

No, in line 159 (now line 161) we are commenting on the lack of a difference between a piliated and non-piliated isogenic mutant in adherence to mNLs. We see no difference in adherence between the piliated and non-piliated strains as mNLs, which unlike hNF, lacks naturally-acquired S-IgA to the pneumococcal type-1 pilus.

*3. In the mouse, the protein Muc5ac is noted with lowercase letters.*

Corrected

*4. Line 335 needs a citation, following “rather than native mucus”. Many groups are studying native mucus.*

Added citations

*5. A statement regarding food and water, temperature, and light cycle of the animal facility would aid the methods section.*

Added information in methods section

*Reviewer #2: General Comments:
1. On line 331, the sentence that reads: “…often found in embedded in the …” needs editing.*

Edited

*Reviewer #3: Please provide in the manuscript the logic for the statistical tests selected for different experiments.*

We made changes so that the statistical tests used to examine the data in different parts of the manuscript are now consistent. Our overall approach is now described in detail in the Statistical Methods section of the Methods (line 852). “A one-way ANOVA, using Dunnett’s multiple comparison test or a Kruskal-Wallis test with Dunn’s multiple comparison test, was used to measure differences in means of 2 or more groups. An un-paired T-test was performed to compared two data sets that passed normality tests, while a Mann-Whitney test was used if the data did not pass normality tests. A sample t and Wilcoxon test was employed to compare data to a theoretical mean of 1, where noted.”

In addition, each figure legend describes the statistical test applied to comparisons.

*6B is misleading – the different time points are done with different animal models and should be plotted separately or at least the distinction made clear.*

As suggested, we divided Figure 6B into two panels (adult and infant) and added additional data/time points. The conclusion is unchanged – there was no significant contribution of *nanA,nanB* to colonization density across time points in either adult or infant mice.

Conclusions and interpretation:

A couple of observations would benefit from more discussion. What do the Authors propose to occur in isolates that naturally lack nanB (and nanC?). Furthermore, is the Authors explanation for the decreased colonization only in co-infection. I am struggling to generate a reasonable hypothesis to explain that.

We added to the discussion (lines 455) that “In isolates that lack *nanB* (4%), Spn may be able to scavenge free 2,7-anhydo-Neu5Ac released by other URT colonizers. If these strains still contain the rest of the *nan* II operon, they could internalize Neu5Ac to activate *nanR* and the transcription of *nanA*. So, even in the absence of *nanB*, the *nan* locus is hypothesized to be able to scavenge free 2,7-anhydo-Neu5Ac to promote *nanA* expression.” We had previously noted in the discussion that “Some strains also encode for NanC, which has many features in common with NanB.” As we have not examined the role of NanC or the rare strains lacking NanB , it seems too speculative to say more.

Regarding co-infection, we discussed two scenarios. 1) In the case of co-infection with influenza A, the virus stimulates mucus production and so the colonization defect of a *nanA* mutant becomes more apparent due to its increased clearance by mucus. 2) In a mixed infection (as in the case of a Tn-Seq screen) of WT and *nanA* mutant Spn, the WT stimulates mucus production and, similar to viral co-infection, this leads to clearance of the mucus-binding *nanA* mutant. In both these scenarios a colonization deficit for the *nanA* mutant is revealed.

Figure 5B. While the rest of the figure is convincing. It is not clear why the nanA complement does not complement the and no removal of sialic acid is observed. This should be discussed, but does not appear to be mentioned in the text at all.

There is no statistically significant difference (One-Way ANOVA Dunnett’s multiple comparison statistical test) between the parent strain (Type 23F::pilus-1) and complemented mutant (Δ*nanA::nanA*) in contrast to the parent strain v. Δ*nanA* which showed a significant difference.

Figure 6A. do the authors know that with these specific strains the mutant has a temporary advantage in binding to mucin in the animal model (line 292)? No longer time points were reported.

We added an additional 5-day timepoint which shows no statistical difference as compared to the early timepoints. This supports the comments made in line 314. As noted above, we are describing *mucus* interactions.

Figure 7A. The Authors mention that similar numbers of bacteria are seen in the top panel. The figure legend doesn’t mention this, but I assume it is the orange staining and the sialic acid green. If that is the case, can conclusions really be drawn from these images there are some significant differences observed. The CFU counts are informative but based on a single image and no quantification I don’t believe these are.

We agree that the images shown are at too high a magnification to compare numbers of Spn, as these are not uniformly distributed along the respiratory epithelium. As low magnification it is clear there are no differences in bacterial numbers between groups. We modified the description of Fig. 7A and stated “Similar numbers of pneumococci were seen along the mucosal surfaces, based on lower-magnification images corresponding the equivalent density of Spn obtained by quantitative culture” (line 336)

Figure 7B and C: Some of these differences are very small – an image of the blot and the boundaries drawn when assessing intensity would be useful for interpretation. Also, the methods say intensity of bands were measured, but were there bands? The rest of the methods read like a dot-blot.

As suggested, we added a representative immunoblot as supplemental data (Fig. S1) to show the bands obtained using a slot blot manifold apparatus to concentrate URT lavage samples.

While the Authors are right, they saw no contribution of MucBP to nasal fluid (Line 344), I believe this experiment was only done with mouse nasal fluid. If that is the case it seems important to acknowledge that the lack of a phenotype could be due to differences in glycosylation between the mucins of different species.

We added a point stating that the lack of effect with MucBP could be due to glycosylation differences between mice and humans. (line 394)

Writing:

The manuscript is for the most part well written. An exception to this are parts of the methods, the strain names and the figure legends. The majority of the comments relative the legends are included below. But as an example

Figure 4. It is not clear from the legend and figure what data in panels C, D and E are plotted relative to. The panels all say wild-type, but the legend says parental strain for C, nanB for D and nanR for E. Is the fold-change relative to mutants in these genes? This doesn’t seem to match the data. Please clarify and make any necessary changes.

Corrected and clarified Figure 4 legend. Panels C, and D are plotted relative to wild-type (Type 23F::pilus-1) not to mutants in these genes.

The strain names used throughout the paper lack consistency and clarity - the strain names in the strain table and methods don't match the text and mutant names in figures and some of the text give no indication of strain background. This makes it harder to follow. Is it possible to modify this and make the figures and text clearer?

We thoroughly addressed these issues in regards to strain names in both the figure legends and methods.

Minor issues
Line 30: Should it read neuraminidase A not just neuraminidase?

Corrected to neuraminidase A.

What is the 23F strain? What site was it isolated from etc.

Added in additional information about Type 23F isolate.

*Line 102: While the point of this sentence is clear after reading it a couple of times it doesn’t seem to make sense as written. I believe a revision could make it clearer for the reader.*

Rewrote this sentence

Figures: some of the lines indicating which conditions were compared by statistical analysis do not align with the bars – for example 1A the NS line actually falls over the 30 second time point bar on the graph.

Edited

Lines 144-5: I assume the authors looked at unbound bacteria under the microscope? Figure 1E does not show bound bacteria are in longer chains than unbound.

We added a new panel to Fig. 1E showing pneumococci in short chains not binding to a mucus in mNLs to help demonstrate the point about chain length.

Line 180: Is NanA present in all strains of nanA?

Clarified sentence

Line 197: I assume the Type 23F strain naturally lacks the pilus locus? Perhaps that could be more clearly communicated.

Added in this information

Line 248: Should it be functional in place of function?

Changed

Line 487: Is this Table S1?

Yes, made the change

Line 626: Doesn’t make sense as written.

Edited

Line 656: Is this strain a pilus deletion or insertion?

Pilus insertion- changed name

Line 893: followed by not following?

Changed

Figure 5A: It might just be me, but it took a long time for me to get my head around this; although, the concept is simple. Is there another way of reporting the data or describing it in the text that might make it easier for the reader to understand the Authors point?

Added additional descriptions to the text to make it easier for the reader to understand the point

Legend figure 7: The figure legend doesn’t mention this, but I assume the orange staining are the bacteria and the sialic acid green. The legend reads as if immunoblots are shown. Furthermore, it says 2,6 linked sialic acid is shown in panel B, whereas the axis says 2,3. Which statistics are applied in each case are also not reported.

Made all changes and clarifications.

Figure 7. The Authors mention that similar numbers of bacteria are seen in the top panel.

See response above on this point.
